# Automated and quantitative characterization of benthic habitat and associated biological communities of an unknown southeast Pacific seamount

**DOI:** 10.1371/journal.pone.0345687

**Published:** 2026-09-15

**Authors:** Yakufu Niyazi, Denise J. B. Swanborn, Jan M. Tapia-Guerra, Javier Sellanes, Erin E. Easton, Germán Zapata-Hernández, Heather A. Stewart, Alan J. Jamieson

**Affiliations:** 1 Minderoo-UWA Deep-Sea Research Centre, School of Biological Sciences and Oceans Institute, The University of Western Australia, Perth, Australia; 2 Programa de Doctorado en Biología y Ecología Aplicada, Universidad Católica del Norte, Coquimbo, Chile; 3 Departamento de Biología Marina, Facultad de Ciencias del Mar, Center for Ecology and Sustainable Management of Oceanic Islands, Universidad Católica del Norte, Coquimbo, Chile; 4 Facultad de Ciencias del Mar, Sala de Colecciones Biológicas, Universidad Católica del Norte, Coquimbo, Chile; 5 School of Earth, Environmental and Marine Sciences, University of Texas Rio Grande Valley, Edinburg, Texas, United States of America; 6 Marine Ecosystems Institute, University of Texas Rio Grande Valley, Port Isabel, Texas, United States of America; 7 Genoa Marine Centre (GMC), Stazione Zoologica Anton Dohrn - National Institute of Marine Biology, Ecology and Biotechnology, Genova, Italy; 8 Kelpie Geoscience Ltd, Edinburgh, United Kingdom; University of Palermo: Universita degli Studi di Palermo, ITALY

## Abstract

Seamounts are prominent seafloor features that strongly influence geological processes, ocean circulation, and benthic biodiversity. Despite their importance, most seamounts remain unmapped and poorly characterized, particularly in the southeast Pacific Ocean, a region recognized for high marine endemism and ecological isolation. In this study, we present a quantitative habitat characterization of a previously undocumented seamount, informally named Solito Seamount, located between the Nazca–Desventuradas Marine Park and the Juan Fernández Archipelago, within Chile’s Exclusive Economic Zone. The seamount is a conical volcanic edifice approximately 37 km wide, rising >3,500 m above the surrounding abyssal plain. High-resolution multibeam bathymetry and backscatter intensity data were integrated with *in situ* observations from two remotely operated vehicle (ROV) dives (S0643 and S0645) to quantitatively investigate how seamount morphology and substrate distribution influence benthic community patterns. An automated abiotic habitat mapping framework incorporating objective terrain analysis and multivariate statistical techniques, including principal component analysis and clustering, was applied to delineate five distinct mesohabitat types: flat, basal slope, valley, ridge slope, and ridge crest. ROV video transects traversing these habitats revealed five associated substrate types, forming macrohabitats: bedrock, bedrock with sediment veneer, sediment-rock transition, sediment, and biogenic rubble. Outputs were used to examine how environmental heterogeneity structures megafaunal assemblages of Solito Seamount. Multivariate analysis revealed a combined effect of habitat and substrate types on benthic megafaunal assemblages across the depth gradient. These compositional dissimilarities were primarily driven by habitat-forming taxa. In the deeper dive (S0643), dissimilarities were distributed across multiple habitats, with ridge crest characterised by reef-building scleractinian community, whereas the ridge slope hosted mixed antipatharian, gorgonian and actiniarian assemblages. In contrast, the shallower dive (S0645) exhibited simpler patterns with fewer taxa driving variation, with substrate effects more evident in biogenic rubble field dominated by sponges (*Stelletta* sp.). Pronounced biological differences between dives may also represent depth-dependent structuring resulting from differences in oxygen regimes associated with water masses, underscoring the role of oceanographic forcing. This study provides the first quantitative habitat map of this previously undocumented seamount, delivering essential baseline information for this largely unexplored region of the southeast Pacific. The integrated multi-scale geophysical and biological approach presented here offers a robust framework for advancing seamount ecosystem understanding and supporting future biodiversity assessments and conservation planning.

## 1. Introduction

Seamounts are isolated elevations rising more than 1,000 m above the surrounding seafloor, exhibiting substantial variation in depth, morphological characteristics, and environmental setting [1–4]. Their formation and subsequent evolution establish new source-to-sink systems that modify local hydrodynamics and sediment-transport pathways [5–7]. These undersea mountains, primarily formed by volcanic activity, influence oceanographic processes by modifying local circulation through current and topography interactions [8,9]. Their rugged topography disrupts prevailing currents, generating flow acceleration, internal tide formation, enhanced turbulence, and localized upwelling, which redistribute particles, modify thermal gradients, and promote vertical mixing [10–12].Volcanic and hydrothermal activity associated with seamounts plays a key role in generating metal-rich deposits, including polymetallic nodules, hydrothermal sulphides, sulphates, elemental sulphur, and phosphate accumulations [13,14].

Beyond their geologic and oceanographic significance, seamounts are widely recognized as ecological hotspots in the deep-sea, supporting high biological productivity, a diverse range of communities and endemism [15–17]. The geological nature of seamounts, including variations in slope, relief, and substrate type, and the overlying hydrodynamic regime creates localized environmental conditions that play fundamental roles in structuring seafloor habitats and biological communities [18–20]. Flat or gently sloping summit plateaus, such as those on the Great Meteor Seamount in the North Atlantic, provide stable substrates that support mass accumulation of free-living scleractinians (stony corals) on soft sediment habitats [21]. In contrast, steep volcanic flanks enhance bottom currents, restrict sediment accumulation, and promote dense assemblages of suspension feeders as documented at the New England Seamount Chain, where slope, sediment types, and dissolved oxygen are the main factors explaining the distribution of diverse sponge taxa [22]. Observations along the Oahu Island of the Hawaiian Ridge suggested that the depth gradient is critical in structuring benthic communities, with small-scale physiographic variations such as the rugosity and slope playing a minor role [23]. The global distribution of seamounts and their three-dimensional complexity result in hotspots of biodiversity that further provide unreplaceable ecological connectivity [17,24]. Habitat heterogeneity, in turn, drives species distribution, community composition, and ecological interactions, contributing to the high benthic and pelagic diversity observed on seamounts [16,25–28].

**Fig 1:**
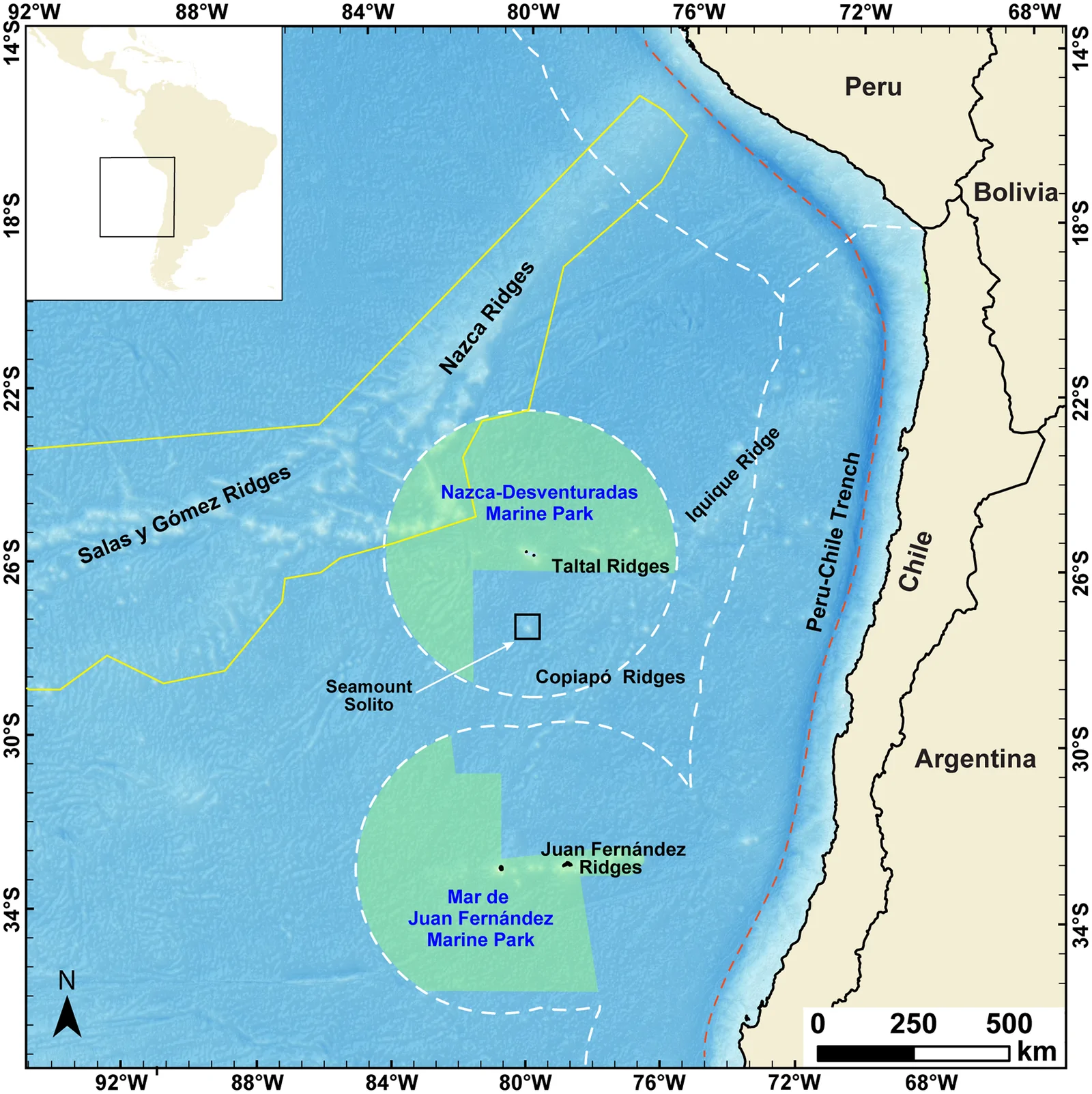
Overview map of the study area in the southeastern Pacific Ocean. The informally named Solito Seamount, the focus of expedition FKt240108, is highlighted by the black box. White dashed lines denote the boundaries of Exclusive Economic Zones (Version 12; https://doi.org/10.14284/632). Green polygons indicate the Nazca-Desventuradas Marine Park and Mar de Juan Fernández Marine Park, and the yellow polygon outlines the Nazca and Salas & Gómez Ecologically or Biologically Significant Marine Area (EBSA) designated under the Convention on Biological Diversity. The red dashed line marks the Peru-Chile Trench, representing the subduction boundary between the Pacific (Nazca) and South American tectonic plates. The basemap was obtained from Natural Earth (http://www.naturalearthdata.com/), and bathymetry is from the GEBCO_2026 Grid, a 15 arc-second global terrain model (https://doi.org/10.5285/4f68d5c7-45eb-f999-e063-7086abc036fa). The inset shows the location of the overview map along the western margin of South America.

Despite their important roles in facilitating enriched ocean biodiversity, only around 4% of global seamounts have been sampled for scientific purposes, with much of our understanding derived from a relatively small number of well-studied sites [29]. Therefore, many seamounts remain unmapped, and quantitative habitat characterization is particularly limited in the southeast Pacific Ocean, where only about one-fifth of the abyssal seafloor has been mapped at high resolution [30]. This lack of systematic exploration poses major challenges for biodiversity assessments, ecosystem modelling, and conservation planning in this region, which hosts numerous seamount chains formed by the Nazca (NZ), Salas y Gómez (S&G), and the Juan Fernández (JF) ridges. This region hosts exceptionally distinctive biological communities, including some of the highest levels of marine endemism recorded globally, and the deepest known light-dependent coral reefs, reflecting a combination of long-term ecological isolation and unique environmental conditions [31–34]. Most seamounts in this region occur along the NZ and S&G ridges, yet only around 3% have been scientifically studied since the 1970s, when expeditions by the former USSR reported high levels of endemism for benthic communities [35–37]. Several scientific campaigns have been conducted in the region, but they have focused almost exclusively on a limited number of ridge-associated features, leaving the vast majority of seamounts poorly characterized or completely unexplored [31,38–40]. To better understand the geomorphological structure, biodiversity, and habitat complexity of these seamounts, several expeditions have been undertaken since 2024. One of which is the Schmidt Ocean Institute’s 2024 expedition FKt240108 (Seamounts of the Southeast Pacific, https://schmidtocean.org/cruise/seamounts-of-the-southeast-pacific/) on board the R/V *Falkor (too)*. During this campaign, an undocumented and formally unnamed seamount (Solito, Fig 1) located between the Nazca-Desventuradas Marine Park and the Juan Fernández Archipelago, within Chile’s Exclusive Economic Zone was surveyed. Solito Seamount is a conical volcanic edifice with a basal diameter of approximately 37 km, rising more than 3,500 m above the surrounding abyssal plain and terminating in a relatively narrow summit at approximately 484 m water depth [41].

Seamounts exhibit strong geomorphological heterogeneity, with geological and oceanographic processes operating across multiple spatial scales, from meso-scale seabed morphological habitats to macro-scale substrate types and benthic community patterns, offering insights into the processes shaping deep-sea ecosystems [42–46]. Consequently, hierarchical habitat characterization frameworks are essential for capturing these multi-scale patterns [47–49]. The first ecological characterization of the Solito Seamount documented the benthic community composition and demonstrated its conservation value [41], yet an assessment linking its morphology, substrate, and biological patterns across multiple spatial scales was lacking. To address this knowledge gap, we adopted an automated quantitative framework that integrates terrain derivatives, acoustic backscatter, principal component analysis, and unsupervised clustering to objectively delineate ecologically meaningful abiotic habitats [48–52]. This approach also addresses several limitations of existing methods, including the Benthic Terrain Modeler and other rule-based classifications that typically rely on user-defined thresholds and expert interpretation that reduced consistency, objectivity, and reproducibility [53–57]. By linking these habitat patterns with macro-scale substrate types and benthic communities documented by ROV surveys, this approach provides a reproducible and scalable framework for seamount habitat characterization and supports robust ecological assessments and conservation planning in data-poor deep-sea environments [45,58,59]

## 2. Materials and methods

### 2.1. Study area

This study focuses on a remote submarine feature, unofficially named Solito Seamount, located in the southeastern Pacific Ocean within Chile’s Exclusive Economic Zone. The seamount lies outside of the Nazca-Desventuradas Marine Park, roughly 200 km south of the Desventuradas Islands and roughly 620 km north of the Juan Fernández Archipelago (Fig 1). Despite its prominence, the tectonic and volcanic origin of Solito remains unresolved. One hypothesis links it to the Iquique Ridge that is currently being subducted beneath the South American Plate [60]. Alternatively, Solito Seamount may represent the westernmost extension of the east–west trending Copiapó Ridge that runs subparallel to the Taltal and JF ridges and is also thought to be subducting beneath the adjacent continental margin [41,61]. However, the limited availability of geochronological and geochemical data for these ridges and Solito constrains efforts to reconstruct their geological evolution. Oceanographically, the southeastern Pacific is structured by vertically stratified water masses that regulate temperature, oxygenation, and near-bottom circulation [62,63]. Surface circulation includes the Humboldt Current and associated coastal branches, supporting high primary productivity along the Chilean coast and continental margin [64,65]. Below surface waters, a persistent oxygen-depleted, nutrient-enriched Equatorial Subsurface Water (ESSW, ~ 50–600 m) is associated with the regional Oxygen Minimum Zone (OMZ). At intermediate depths (600–1,300 m), well-oxygenated Antarctic Intermediate Water (AAIW) flows northward, whereas nutrient-rich and well-oxygenated Pacific Deep Water (PDW; ~ 1,300–3,000 m) occupies deeper levels. Below the PDW, Circumpolar Deep Water (CDW), together with contributions from Antarctic Bottom Water (AABW) and North Atlantic Deep Water (NADW), dominates abyssal circulation [65]. From a biogeographic perspective, Solito occupies a strategic position between the NZ, S&G, and JF ridges, which may form part of a proposed connectivity corridor that may enable faunal exchange between isolated oceanic islands and continental deep-sea ecosystems [31,33,39,40].

### 2.2. Data collection

#### 2.2.1. Multibeam echosounder data.

**Fig 2:**
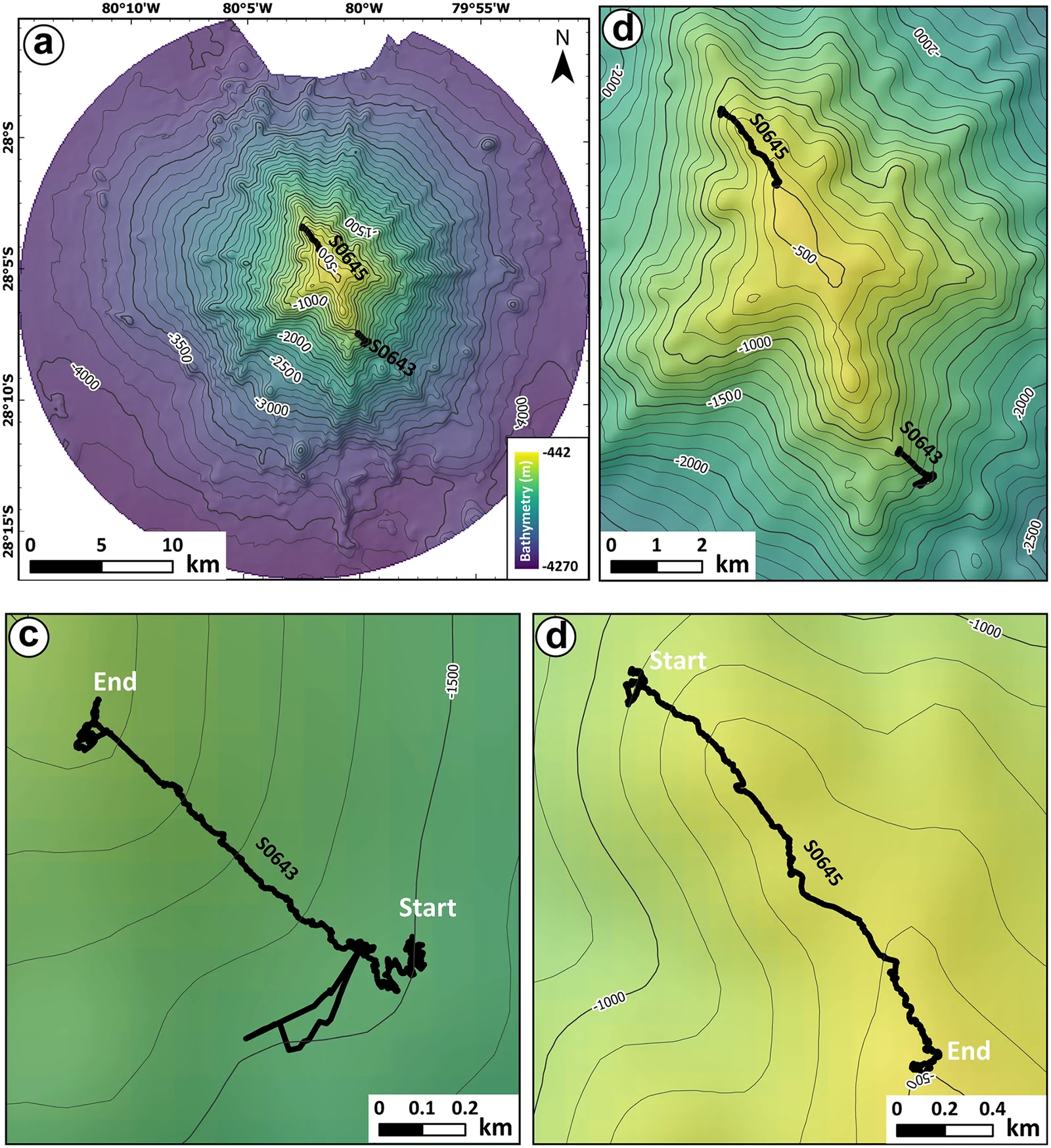
High-resolution bathymetry and ROV dive tracks of Solito Seamount. (a) High-resolution multibeam bathymetry of the Solito Seamount and remotely operated vehicle (ROV) dive tracks (black lines) acquired during expedition FKt240108. (b) Zoom in of the seamount summit and ROV dive locations. Close up ROV dive tracks for (c) S0643 and (d) S0645. Contours at 100 m intervals.

High-resolution bathymetry and acoustic backscatter intensity measurements were collected using a hull-mounted Kongsberg EM 124 multibeam echosounder (MBES). Operating at a nominal frequency of 12 kHz within a 10.5–13.5 kHz range, the system has a beam width of 0.5° × 1° and is optimized for depths between 1,000 and over 8,000 m. Data acquisition was conducted via Kongsberg’s Seafloor Information System (SIS, version 5) with vessel positioning supplied by a Seapath 380 + navigation system. Sound velocity profiles were recorded using a Reson SVP70 sensor attached to the transducer head, complemented, when necessary, by expendable bathythermograph (XBT) drops. All raw data were processed in QPS Qimera software suite (version 2.5) with final bathymetric digital elevation models (DEMs) gridded and exported at a resolution of 100 m pixel size (Fig 2). The FMGeocoder Toolbox (version 7.10) for Fledermaus was used to produce backscatter mosaic at a resolution of 50 m pixel size, depicting acoustic reflectivity patterns.

#### 2.2.2.. ROV data.

The 4,500-m–rated ROV *SuBastian* conducted benthic video surveys using high-definition (HD) and 4K ultra-high-definition (UHD) cameras. The ROV is equipped with two 4K Sulis Subsea Z70 (resolution 840 × 2160 pixels) deep-sea video cameras and four DSPL FlexLink HD Multi SeaCams [66]. Two ROV dives were undertaken on Solito at sites selected based on the location of topographic ridges and hardgrounds, interpretated from the MBES data (Fig 2). These dives were conducted on the southern flank at ~1,054–1,497 m (lower to mid slope dive, S0643) and on the northern flank at ~456–860 m (upper slope to summit dive, S0645) (Table 1, Fig 2). The ROV transects primarily advanced upslope, surveying benthic invertebrates, benthopelagic organisms, and seafloor geology. The surveys yielded approximately 16 h of HD video footage for benthic surveys and specimen collections. Logging of observations and sampling events were made using the Sealog annotation system [67,68], which allowed systematic logging of frame grabs together with environmental and positional metadata. For benthic fauna assessments, frames were captured every 10 s from the videos using the ASNAP (auto snapshot) function in the Sealog software to ensure consistent and reliable frame extraction, providing accurate and unbiased sampling for subsequent abundance analyses.

**Table 1:** Summary of ROV dive operations conducted at the Solito Seamount during expedition FKt240108.

Dive ID	Start of transect coordinates		Length of transect (km)	Depth range (m)	Number of frames per dive for substrate assessments	Number of standardized benthic quadrats per dive
	Latitude (DD)	Longitude (DD)				
S0643	−28.130	−80.003	5.1	1,497–1,054	1395	66
S0645	−28.0704	−80.044	3.8	860–456	1451	68

### 2.3. Data analysis

#### 2.3.1. Quantitative habitat mapping.

This study follows the habitat classification framework of Greene et al. [47,69], whereby the entire seamount is defined as a megahabitat (> 1 km), and the morphological features occurring within the seamount, such as ridges, valleys, and slopes, are classified as mesohabitats spanning spatial scales from tens of metres to several kilometre. To characterise the meso-scale habitats (habitats) across Solito, we first applied quantitative terrain analysis using the multibeam bathymetry and backscatter intensity data gridded at 100 m and 50 m resolution respectively. A suite of seafloor terrain derivatives was calculated from the bathymetric grid to characterize geomorphological complexity. These derivatives include slope, bathymetric position index (BPI), profile curvature, planform curvature, terrain ruggedness index (TRI), and vector ruggedness measure (VRM) (S1Table). Slope and curvature metrics characterize local and along-slope seabed morphology, providing insights into the fine-scale structure of the seafloor. In contrast, BPI, TRI, and VRM quantify broader aspects of topographic complexity and surface heterogeneity. Collectively, these bathymetric derivatives have been extensively applied in both quantitative and qualitative habitat mapping and have demonstrated strong utility in describing the benthic ecosystems [52,53,70–73]. An internal radius of 100 m and an external radius of 300 m were used to calculate fine-scale BPI, while an internal radius of 200 m and an external radius of 500 m were adapted to calculate broad-scale BPI, thereby capturing variations across multiple scales (S1 Fig 1). Backscatter intensity data were incorporated as a measure of relative acoustic reflectivity and included as an input variable in the multivariate habitat classification [74–77]. Importantly, this classification is strictly abiotic in nature, aiming to delineate morphologically and acoustically distinct seafloor habitats rather than biologically defined habitats.

Because the two ROV transects did not span the full depth range of Solito, they provided only limited representative training data for supervised classification. Therefore, a broad-scale habitat classification was performed using established unsupervised methods to identify natural groupings in the bathymetric derivatives and backscatter data [49,51,78]. Principal component analysis (PCA) was performed [79] to reduce data dimensionality and keep the most informative and uncorrelated components to capture the majority of variance in the dataset [80]. Variables were scaled and centred before conducting the PCA to ensure they had equal weight in the analysis. To classify seafloor habitats, k-means clustering was applied to the retained principal components (PCs) [81]. The optimum number of clusters was determined via an elbow plot. The characteristics of each cluster were evaluated using boxplots of the PCA scores [82]. The resulting clusters were mapped back onto the original bathymetric surface, producing a spatially explicit representation of benthic habitats as defined by broad-scale geomorphology [46,47,83–85]. All quantitative habitat mapping analyses were conducted using the open-source software R (version 4.4.2) [86] and the packages *raster*, *RStoolbox*, *terra*, *reshape2*, *MultiscaleDTM*, and *spatialEco* [87–89].

#### 2.3.2. Macro-scale substrate characterisation from ROV imagery.

To provide macro-scale substrate information, ROV video data were used to classify substrate types throughout the dives. Each ROV video transect was first reviewed in full to gain a general understanding of the substrate variability and morphological context. Video frames were exported as still images at 30-second intervals to balance spatial representativeness with analytical efficiency [90], generating a total of 2,846 benthic still images for review. Images with low visibility (due to sediment or lighting) were excluded. Substrate types were classified according to the relative occurrence and predominance (>80%) of three main components: bedrock, sediment, and biogenic material. This classification was conducted through quantitative visual interpretation of seabed imagery, by comparing and adapting established substrate classification frameworks that are widely used in marine habitat studies [47,83,91–94]. Each image was assigned to one of the defined substrate classes with the predominant substrate composition, sediment cover, and structural complexity examined.

#### 2.3.3. Benthic fauna assessments.

A subset of ROV ASNAP video frames were established as quadrats and were defined based on the following criteria: (i) a field of view between 1 and 5 m² to ensure accurate species identification and (ii) frames in which the ROV maintained a steady altitude between 1 and 5 m above the substrate, ensuring the visibility of the laser scalebar (two points positioned 10 cm apart) for precise measurement. Of these frames, a total of 134 frames, one per every ~15 min of ROV dive time, were used for the standardized photographic quadrats (1–5 m²) [41], comprising 66 from S0643 and 68 quadrats from S0645 (Table 1). Note that the dive transects were primarily focused on benthic (sessile and mobile) and benthopelagic organisms, thus fish were not considered in this research because no standardized method for fish abundance was used during data acquisition. Taxa were identified to the lowest possible taxonomic level based on regional taxonomic literature and previous faunal inventories from the Nazca–Desventuradas Marine Park and the Juan Fernández Archipelago [95–100], complemented by specimens collected during the present expedition. Specimens were collected using the ROV manipulator, preserved in 96% ethanol, and deposited in the Sala de Colecciones Biológicas of the Universidad Católica del Norte (SCBUCN) for detailed laboratory examination. Specimen collection and handling were conducted under permit R. EX. Nº E-2023–612, issued by the Chilean National Fisheries and Aquaculture Service (SUBPESCA) to Universidad Católica del Norte, Chile. All identifications were subsequently cross validated using authoritative global databases, including the World Register of Marine Species (www.marinespecies.org) and the Ocean Biodiversity Information System (www.obis.org)

#### 2.3.4. Statistical analysis.

A hierarchical framework was used to evaluate the structuring effect of multi-scale habitat characteristics on benthic biodiversity patterns at Solito. Differences in benthic assemblage structure between habitat classes and substrate types were first assessed using a Permutational Multivariate Analyses of Variance (PERMANOVA) in PRIMER version 7.0 on a Bray-Curtis dissimilarity matrix of square-root-transformed data and over 9,999 permutations. Dive was included as a random factor, habitat as a fixed factor nested in dive, and substrate as a fixed factor nested in habitat and dive. Follow-up pairwise tests were used to assess which habitat types and substrates significantly differed based on benthic assemblage structure.

To identify the taxa contributing most strongly to compositional differences among habitat and substrate types per dive, Similarity Percentage Analyses (SIMPER; [101]) were applied in R (version 4.4.2) [86] using package *vegan* [102] and *tidyverse* [103]. The dive community datasets were cleaned to exclude empty taxa (columns with zero counts), empty samples (rows with zero counts), and rare taxa (occurring in < 2 frames). SIMPER analyses were performed over 9,999 permutations on the cleaned community matrices using Bray-Curtis dissimilarity. SIMPER analyses were conducted separately for each dive to avoid confounding habitat-substrate contrasts with between-dive differences. Only habitat-substrate combinations represented by at least two replicate frames were retained for the analysis. SIMPER was performed for habitat (e.g., ridge crest and ridge slope), substrate (e.g., bedrock and sediment) as well as a combined habitat and substrate factor (e.g., ridge crest-bedrock and basal slope-sediment) per dive. SIMPER outputs were tidied and combined across dives by extracting the taxa contributing significantly (p < 0.05) to group dissimilarities for each contrast. Taxa were assigned to these groups to identify taxon-habitat associations within each dive. Contributions were aggregated to create heatmaps of taxa most strongly associated with (1) each habitat grouping, (2) each substrate grouping, and (3) each combined habitat-substrate grouping.

## 3. Results

### 3.1. Seamount habitats

**Fig 3:**
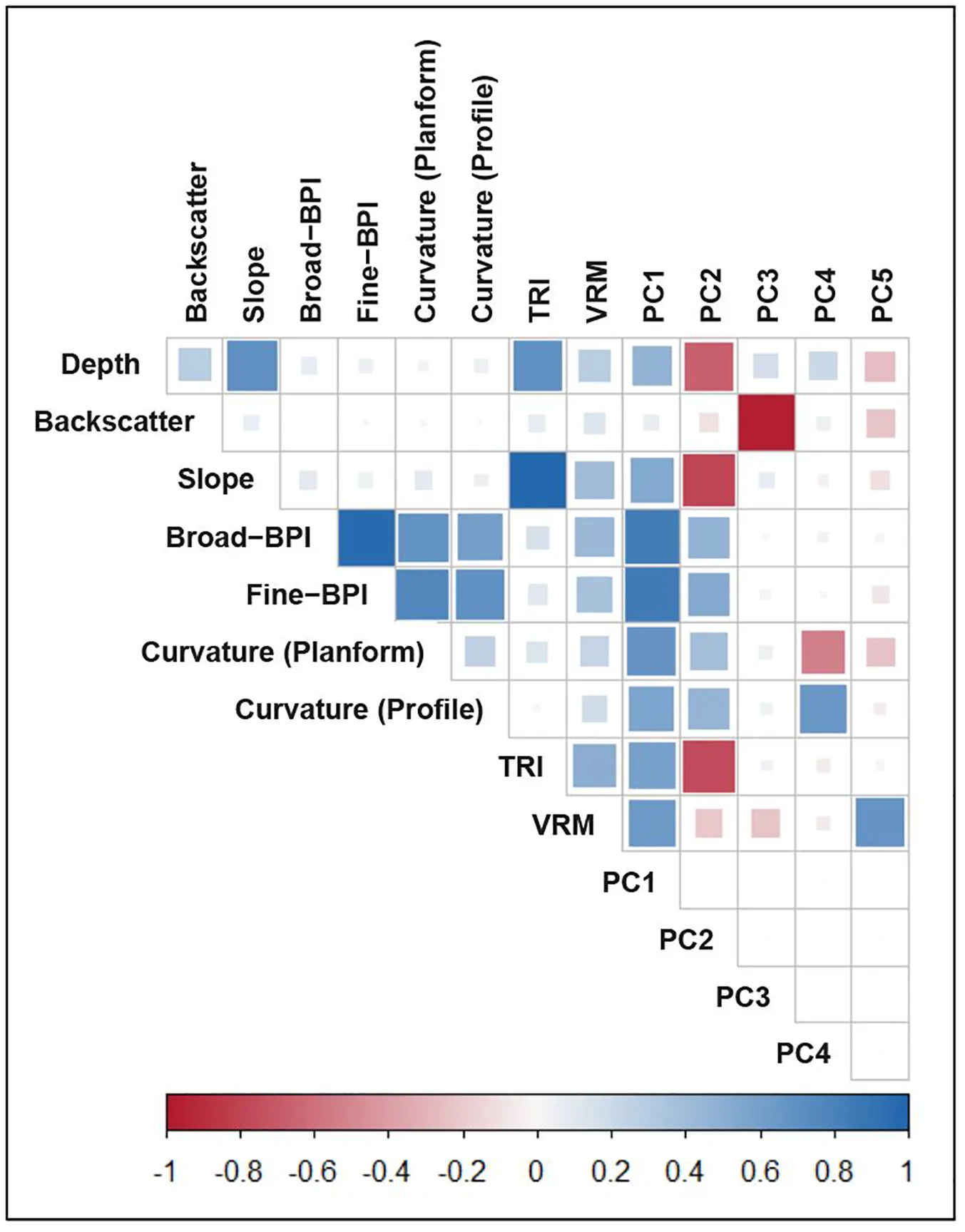
Correlation heat map of depth, backscatter intensity, slope, BPI, curvatures, TRI, VRM and main principal components. BBPI and FBPI: broad and fine-scale bathymetric position index; TRI: terrain ruggedness index, VRM: vector ruggedness measure.

The PCA of the nine abiotic variables revealed clear groupings among morphological, terrain, and acoustic predictors (S1 Fig, S1 Table) with the five PCs explaining 93.9% of the total variance. The first two components explained a combined 67% of the variance (38.4% and 28.6%, respectively), indicating they capture most of the essential structure in the data (S2 Fig). The inclusion of the third, fourth, and fifth components substantially increased the cumulative variance, indicating that these components represented additional but still meaningful gradients. The marked decline in explained variance beyond the fifth component suggested that higher-order components contribute little additional information and were therefore excluded from further analysis. The rotated component matrix showed the factor loadings that explain the correlations between the rotated PCs and the original variables (S2 Table). Bathymetric position and curvature metrics (broad-BPI, fine-BPI, planform curvature, and profile curvature) loaded strongly on the first component, indicating a dominant geomorphological gradient separating crests, flanks, and depressions (Fig 3, S2 Table). Slope and TRI were the primary contributors to the second component, reflecting gradients in steepness and relative topographic relief, whereas backscatter intensity overwhelmingly defined the third component, isolating a substrate reflectivity signal (S2 Table). VRM and curvature metrics contributed to higher-order components, capturing fine-scale surface complexity. Correlation patterns (Fig 3) further indicated a strong negative relationship between depth and backscatter intensity, suggesting lower acoustic reflectivity in deeper areas, and a positive association between slope and TRI. Overall, the retained PCs summarised the major physical drivers of habitat heterogeneity on Solito Seamount and provided an ecologically interpretable, low-collinearity set of predictors for subsequent habitat mapping analyses.

**Fig 4:**
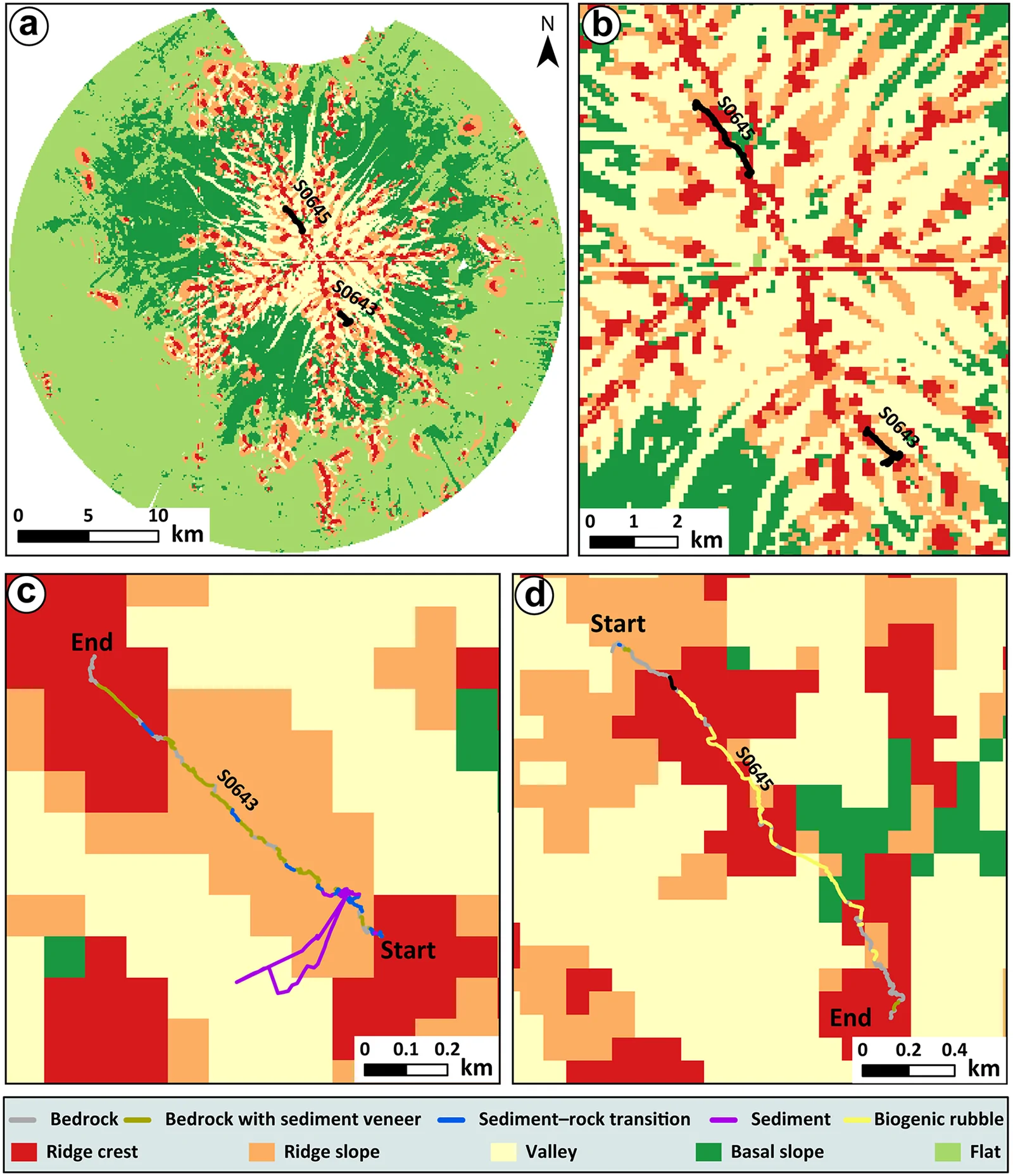
Habitat classification and ROV observations of Solito Seamount. (a) Habitat classification for Solito Seamount and remotely operated vehicle (ROV) dive tracks (black lines). (b) Zoom in of the habitat classification for the seamount summit with ROV dive locations (black lines). Close up ROV dive tracks with observed substrate types for (c) dive S0643 and (d) dive S0645, overlain on habitat classification for the whole seamount.

The k-means clustering analysis, with five clusters selected using the elbow method, revealed distinct and spatially coherent habitat patterns across the seamount (Fig 4). Violin–boxplots of standardized of geomorphometric and acoustic variables showed clear differences among the five habitat classes, and allowed these classes to be associated with five morphological structures of the seamount ([1] ridge crest, [2] ridge slope, [3] basal slope, [4] valley, and [5] flat seafloor) (Fig 4, S3 Fig), indicating that the classification effectively captured the major variations in seafloor morphology and acoustic response. Habitat 1 was one of the most distinct habitat classes, interpreted as seamount ridge crests, characterized by relatively shallow depths, strongly positive BPIs, elevated TRI and VRM, and generally moderate to high slopes. These attributes were consistent with topographically elevated, structurally complex localized ridge crests (S3 Fig). Habitat 2 was also associated with elevated and structurally complex terrain but differs from ridge crest by occurring on steeper slopes with slightly greater depths and lower BPI values (S3 Fig). Habitat 3 (basal slope) occured intermediate depths and was characterized by moderate slopes, slightly negative BPI values, and comparatively low ruggedness metrics. These characteristics were consistent with lower flank settings that formed a geomorphic transition between steeper ridge slope and adjacent low-relief terrain (S3 Fig). Habitat 4 (valley) occurred at greater depths and was characterized by negative BPI values, gentle slopes, low backscatter intensity, and reduced terrain ruggedness, indicating depressed topographic settings that likely promote sediment accumulation and smoother substrate conditions (S3 Fig). Habitat 5 (flat seafloor) represented extensive low-relief areas surrounding the seamount. It was characterized by greater depths, minimal slopes, near-zero curvature, low TRI and VRM values, and generally low acoustic backscatter, consistent with smooth, laterally extensive abyssal to near-abyssal plain environments (S3 Fig).

### 3.2. Substrate types and distribution

**Fig 5:**
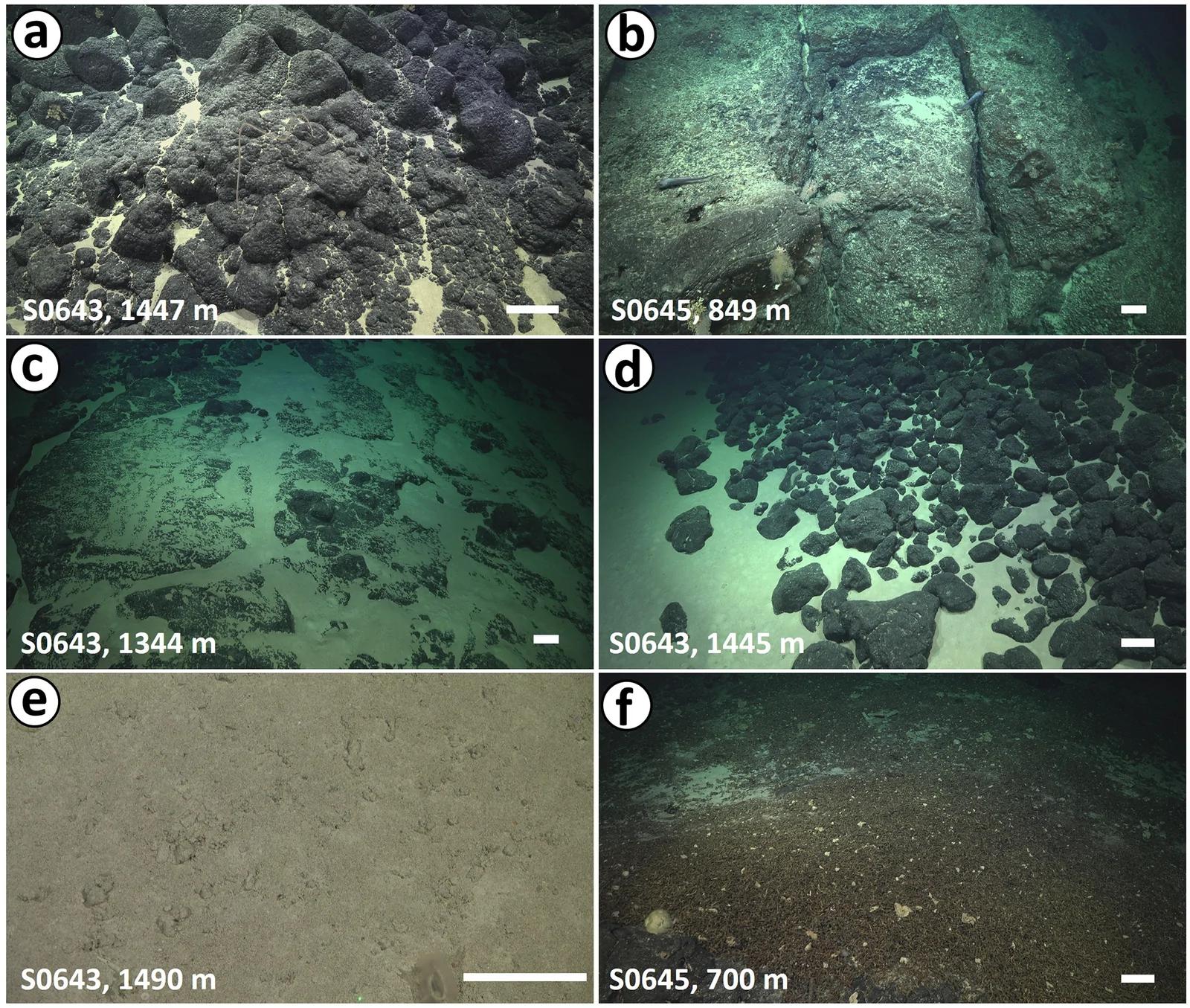
Examples of classified substrate types along the remotely operated vehicle (ROV) dive tracks. (a) and (b) are bedrock, (c) bedrock with sediment veneer, (d) sediment–rock transition, (e) sediment, and (f) biological rubble. The white scale bar represents 10 cm. Images are from Schmidt Ocean Institute [66].

**Fig 6:**
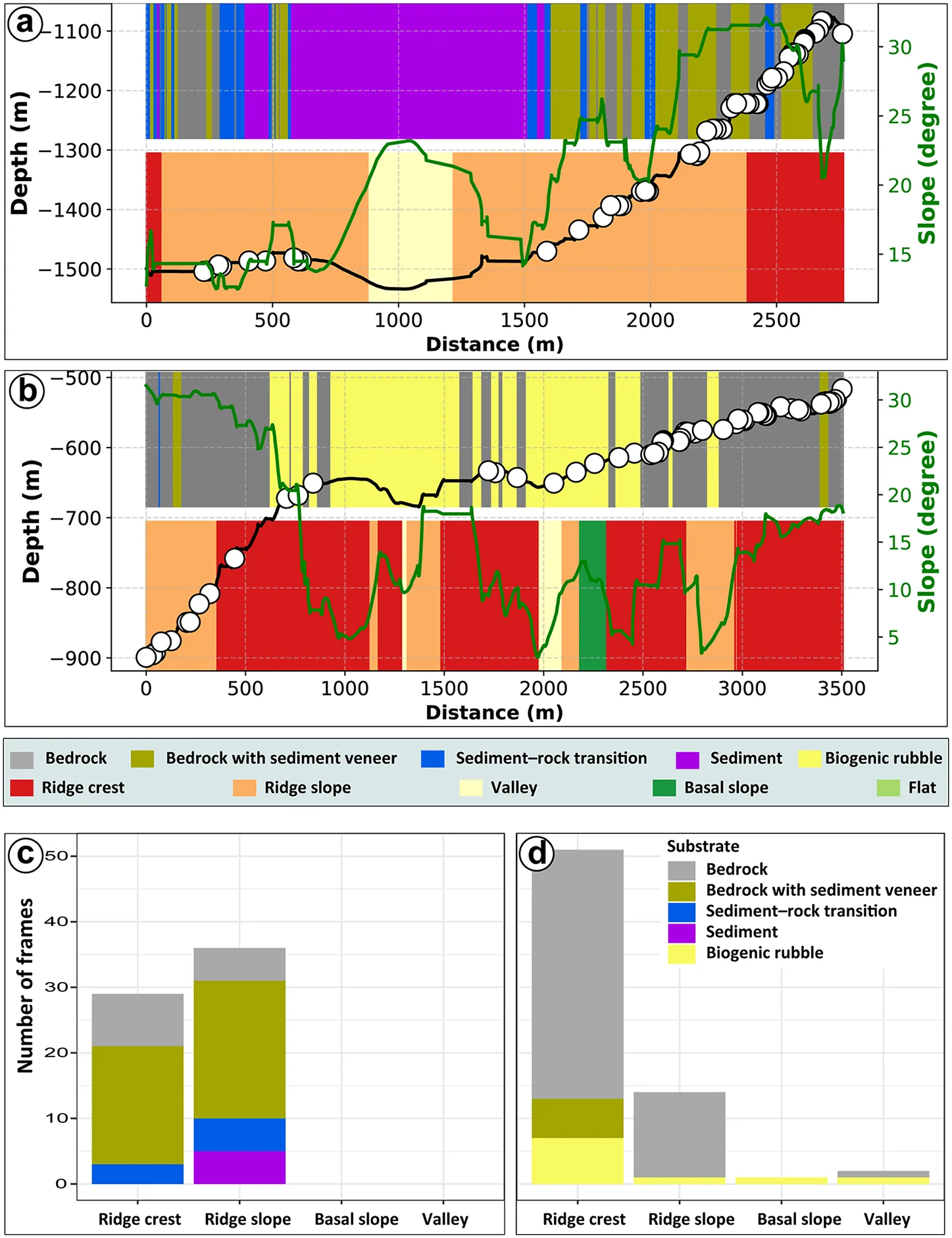
Spatial visualisation of quantitative habitat and substrate types identified on Solito Seamount. Depth (black line) and slope (green line) profiles along the two remotely operated vehicle (ROV) dive tracks with mesohabitats (lower half) and substrate types (upper half) for (a) dive S0643 and (b) dive S0645. Depth profile showing only locations where the seafloor was visible and clearly resolved in the ROV video footage. White circles represent biological observations. Number of frames of substrate types observed in different habitats along ROV dive S0643 (c) and dive S0645 (d).

Five substrate types were identified along the ROV transects: bedrock, bedrock with sediment veneer, sediment–rock transition, sediment, and biogenic rubble (Table 2; Figs 4–6). Outcropping bedrock consisted of exposed volcanic lava flows with rough surfaces, high structural complexity, and little to no (<5%) sediment cover. Bedrock with sediment veneer comprised volcanic bedrock partially draped by a thin (5–20%) layer of sediment (Table 2; Fig 5). Sediment–rock transition substrates formed heterogeneous mosaics of exposed bedrock, talus cobbles, and unconsolidated sediments, exhibiting moderate to high small-scale heterogeneity and variable sediment cover (10–50%). Sediment substrates were dominated by unconsolidated coarse-grained material forming smooth, low-relief seafloor with low structural complexity and high (>80%) sediment coverage. Biogenic rubble consisted of accumulations of fragmented coral skeletons and skeletal debris mixed with sediment, displaying low to moderate structural complexity and variable (20–50%) sediment coverage.

**Table 2:** Substrate types, their dominant substrate composition, structural complexity and sediment coverage observed along the ROV dive tracks of the Solito Seamount.

Substrate type	Description	Dominant substrate composition	Structural complexity	Sediment coverage
Bedrock	Massive volcanic outcrops or crusts forming hard, rugged surfaces with little or no sediment accumulation.	Lava flows, pillow lavas, boulders or cobbles	High	Absent (<5%)
Bedrock with sediment veneer	Volcanic bedrock outcrops partially draped by a thin veneer of sand or silt; sediment accumulates in cracks or depressions.	Lava flows, boulders or cobbles	High	Thin (5–20%)
Sediment–rock transition	Mixed mosaic of exposed volcanic rock, cobbles, and interstitial sediments; exhibit high occurrence of small-scale heterogeneity.	Sediments and bedrocks or cobbles	Moderate to high	Variable (10–50%)
Sediment	Uniformly distributed unconsolidated soft sediment forming a smooth or rippled seabed with low relief; no visible bedrock.	Unconsolidated sediments, mostly coarse grained	Low	High (>80%)
Biogenic rubble	Accumulations of dead coral fragments, skeletal debris, and shells, often mixed with sediment and occasionally overlying hard substrate.	Biogenic remnants and sediments	Low to moderate	Variable (20–50%)

Both ROV dives traversed multiple seamount habitats and substrate types, with S0643 encountering a greater diversity of substrate types than S0645 (Figs 4 and 6). However, the overall substrate composition differed between the two transects. ROV dive S0643 showed relatively heterogeneous substrates (Figs 4c and 6). Bedrock with sediment veneer accounted for roughly 60%, and exposed bedrock constituted approximately 20% of all frames observed in dive S0643 (Fig 6c). While sediment–rock transition and sediment substrates each contributed about 10% of observations. In contrast, S0645 was strongly dominated by exposed bedrock, which comprised >80% of all frames (Fig 6d). Biogenic rubble formed a minor component (~12%), and bedrock with sediment veneer accounted for <10% of observations. Sediment and sediment–rock transition substrates were absent along this transect.

Across both dives, substrate distribution closely corresponded with seafloor habitats (Figs 4 and 6). Ridge crest and ridge slope were consistently dominated by hard substrates (bedrock and bedrock with sediment veneer), reflecting minimal sediment accumulation, whereas valley and basal slope favoured softer sedimentary substrates with lower structural complexity (Fig 6). At S0643, ridge crest habitat was dominated by bedrock with sediment veneer (~60–65%), with exposed bedrock (~25–30%) and minor sediment–rock transition (~10%). Ridge slope was more heterogeneous, with bedrock with sediment veneer remaining dominant (~55–60%) and exposed bedrock, sediment–rock transition, and sediment each contributing ~10–15%. The valley habitat was characterized by smooth, low-relief seafloor composed mainly of unconsolidated sediments (Fig 6a). At S0645, ridge crest and ridge slope were overwhelmingly composed of exposed bedrock (~70–90%), while the biogenic rubble occurred locally (~10–15%). The basal slope habitat was also sparsely represented and consisted mainly of biogenic rubble.

### 3.3. Benthic taxa and multi-level habitat associations

#### 3.2.1. PERMANOVA.

Predominant benthic taxa observed along the dive transects are listed in Table 3. Results of the PERMANOVA demonstrated that dive, habitat (nested within dive), and substrate (nested within habitat and dive) all contributed significantly to variation in benthic community structure. A significant effect of dive was detected (Pseudo-F = 2.02, p = 0.008; S3 Table), likely reflecting unmeasured variation between dives, such as depth or oceanographic differences. Habitat nested within dive explained additional variation (Pseudo-F = 1.64, p = 0.001; S3 Table), indicating that different geomorphological habitat classes within a dive contain different assemblages. The effect of substrate nested within habitat was strongest (Pseudo-F = 2.19, p = 0.001; Fig 8, S3 Table) and contributed the most to the variance explained, indicating that substrate explained the greatest proportion of variation in benthic community composition. Pairwise comparisons of habitat within each dive showed that habitat effects were not consistent across dives. In S0643, benthic assemblages differed significantly between ridge crest and ridge slope habitats (t = 1.40, p = 0.005; Fig 8, S4 Table). In contrast, no habitat pairs were significantly different within S0645, although several comparisons could not be tested due to insufficient replication (S4 Table). Pairwise tests of substrate nested within habitat and dive confirmed strong but variable effects of substrate. In S0643, significant differences were detected among several substrate types within the ridge slope, with sediment-associated communities being consistently different from those on other substrate types (p < 0.01) (S5 Table). Weaker differences between substrate types were found in the ridge crest in the same dive. In SO465, conversely, substrate differences were most pronounced within the ridge crest, where biogenic rubble differed significantly from both bedrock and sediment veneer. Limited replication meant not all combinations could be tested.

**Table 3:** Dominant benthic organisms observed along the ROV dive transects of the Solito Seamount (for full descriptions of the benthic community see Tapia-Guerra et al. [41]). Numeric label refers to their representation of SIMPER contributions in Fig 7.

Numeric Label	Phylum	Class	Order	Family	ID
1	Porifera	Demospongiae	Tetractinellida	Ancorinidae	*Stelletta* sp.
2	Echinodermata	Echinoidea	Pedinoida	Pedinidae	*Caenopedina* sp.
3	Echinodermata	Echinoidea	Aspidodiadematoida	Aspidodiadematidae	*Aspidodiadema* sp.
4	Echinodermata	Crinoidea	Comatulida	Antedonidae	Antedonidae sp.
5	Cnidaria	Octocorallia	Stolonifera	Alcyoniidae	*Anthothela* sp.
6	Cnidaria	Hexacorallia	Scleractinia	Dendrophylliidae	*Enallopsammia rostrata*
7	Cnidaria	Hexacorallia	Scleractinia	Caryophylliidae	*Madrepora oculata*
8	Cnidaria	Hexacorallia	Scleractinia	Caryophylliidae	*Caryophyllia* sp.
9	Cnidaria	Hexacorallia	Antipatharia	Schizopathidae	*Telepathes* sp.
10	Cnidaria	Hexacorallia	Antipatharia	Schizopathidae	*Bathypathes* sp.
11	Cnidaria	Octocorallia	Scleralcyonacea	Primnoidae	*Calyptrophora* sp.
12	Cnidaria	Octocorallia	Scleralcyonacea	Plexauridae	Plexauridae spp.
13	Cnidaria	Octocorallia	Scleralcyonacea	Keratoisididae	*Acanella* sp.
14	Cnidaria	Octocorallia	Scleralcyonacea	Coralliidae	*Hemicorallium* sp.
15	Cnidaria	Octocorallia	Scleralcyonacea	Chrysogorgiidae	*Chrysogorgia* sp.
16	Cnidaria	Hexacorallia	Actiniaria	Actinoscyphiidae	*Actinoscyphia* sp.
17	Arthropoda	Malacostraca	Decapoda	Nematocarcinidae	*Nematocarcinus* sp.

**Fig 7:**
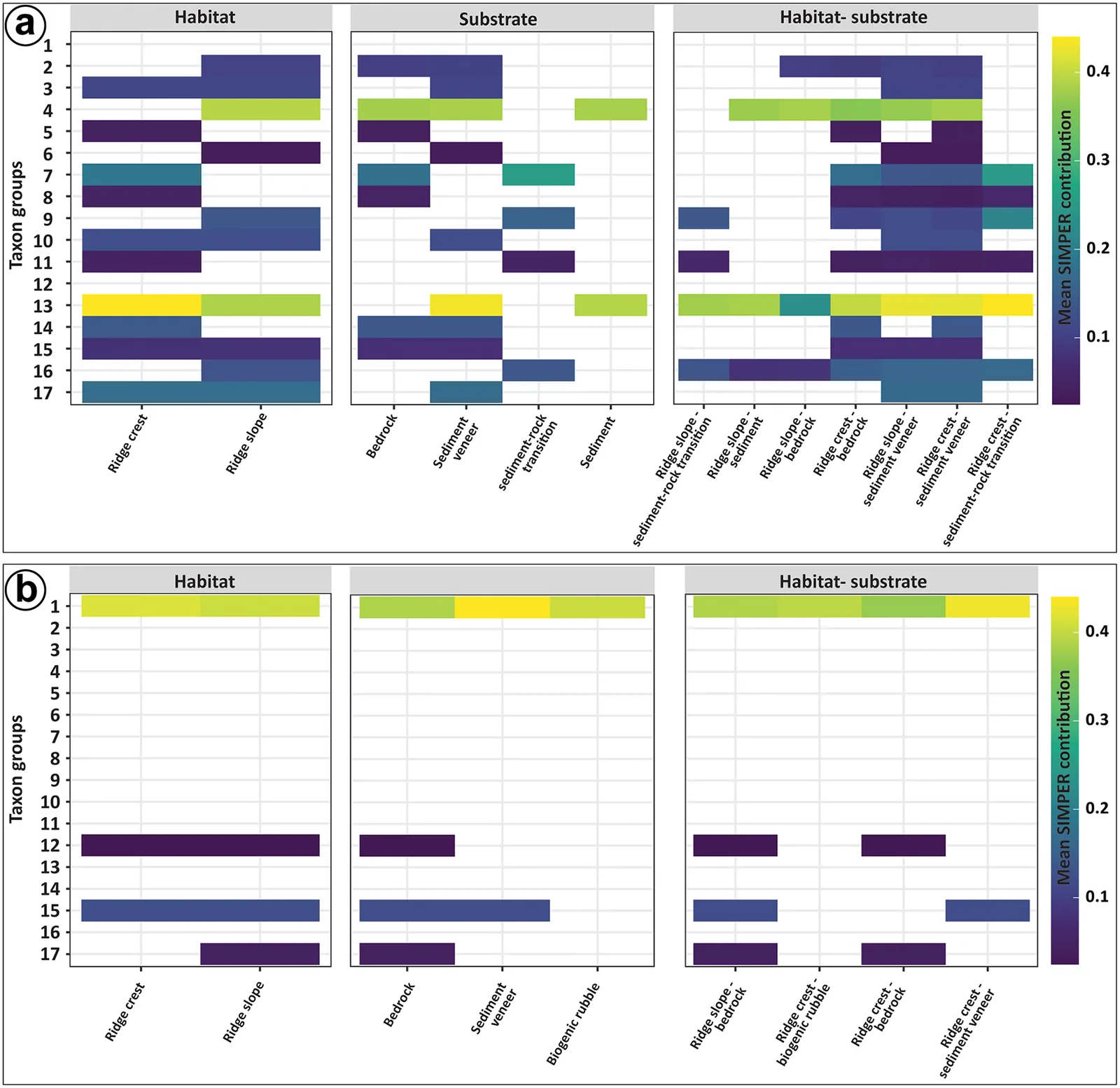
Distribution of faunal groups within the different habitats (left), substrates (middle) and combination of habitats and substrates (right), along remotely operated vehicle dive (a) S0643 and (b) S0645. Taxa groups are displayed in Table 3.

The number of taxa contributing significantly to compositional differences differed between dives. For dive S0643, SIMPER identified a broad suite of taxa contributing to habitat, substrate and combined habitat-substrate contrasts, including reef-building scleractinians, octocorals, antipatharians, actiniarians, echinoderms and sponges (Fig 7). In contrast, dive S0645 exhibited fewer taxa that contributed significantly to assemblage dissimilarities. The main contributing taxa were the sponge *Stelletta* sp*,* the gorgonians *Chrysogorgia* sp. and Plexauridae spp., and the shrimp *Nematocarcinus* sp., often with one or two taxa accounting for the majority of the dissimilarity.

#### 3.3.2. Habitat associations (habitat-only SIMPER).

When taxa associations were assessed per habitat (Fig 7), several topography-linked patterns were evident, being the most pronounced in dive S0643. Dissimilarities among S0643S0643 ridge crest and all other habitats were predominated by reef-building and erect suspension feeders, including *Solenosmilia* sp.*, Caryophyllia* sp.*, Enallopsammia* sp.*, Calyptrophora* sp.*, Chrysogorgia* sp. and *Hemicorallium* sp*.* This predominance reflected a distinct summit assemblage characterized by hard-bottom reef-forming and branching taxa. The S0643S0643 ridge slope habitat supported a mixed assemblage of antipatharians (*Telopathes* sp.), gorgonians (*Acanella* sp.), actiniaria (*Actinoscyphia* sp.)*,* and crinoids (Antedonidae sp.). Associations for S0643S0643 basal slope and valley habitats could not be reliably resolved due to low replication. In dive S0645, habitat similarity was higher than in S0643 with a more restricted set of taxa showed meaningful habitat associations. Octocorals Plexauridae sp., *Chrysogorgia* sp.*,* and the sponge *Stelletta* sp. were representative of S0645 ridge crest and ridge slope habitats. Nematocarcinid shrimp (*Nematocarcinus* sp.) differentiated S0645 ridge crest and ridge slope habitats, even where substrate types were identical, suggesting an association with geomorphological setting beyond substrate type substrate in S0645.

#### 3.3.3. Macro-scale substrate associations (substrate-only SIMPER).

**Fig 8:**
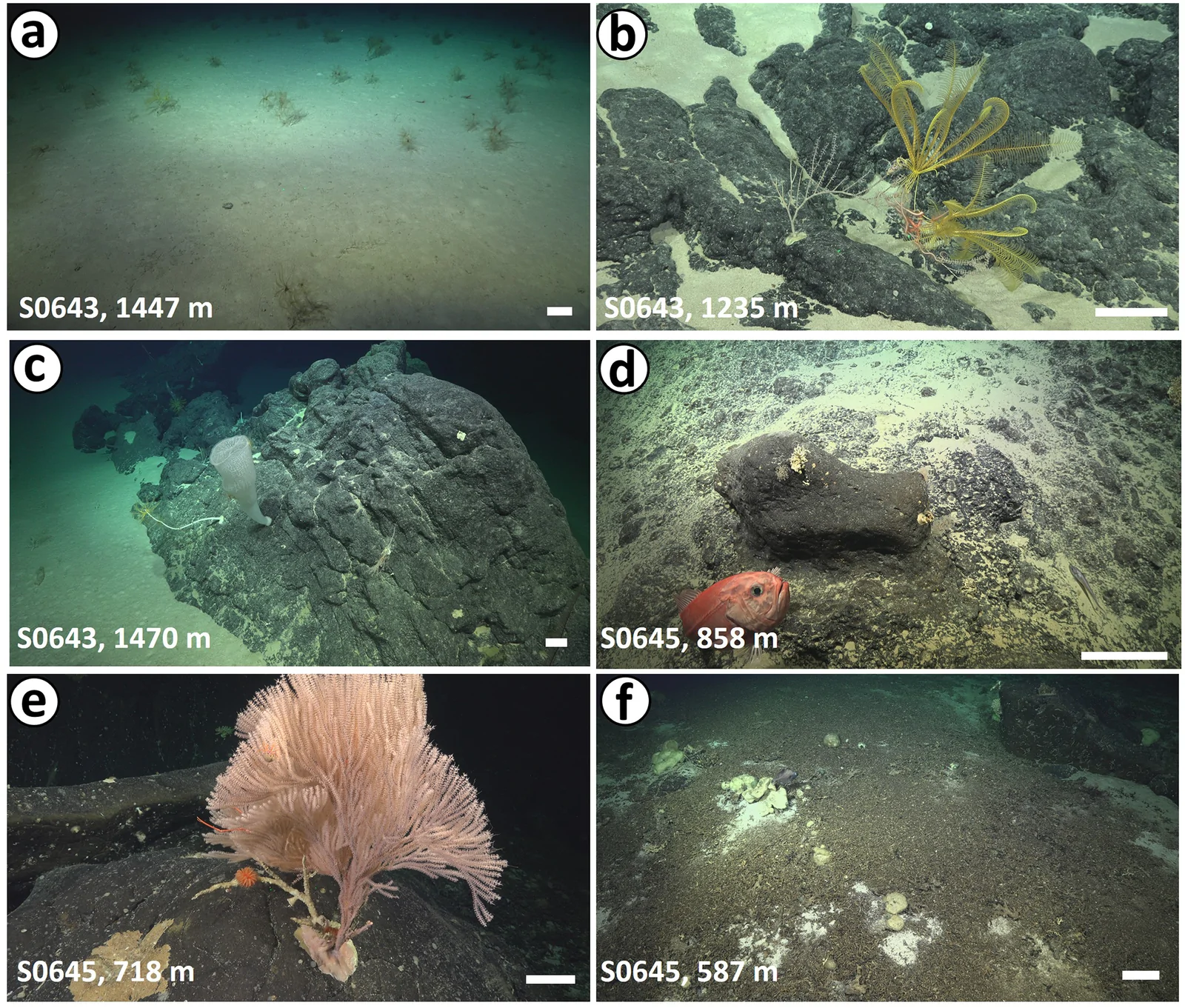
Representative examples of megafauna observed on different substrate types. (a) Fields of small bamboo corals (*Acanella* sp.) and an antedonid crinoid on soft sediment. (b) Antedonid crinoid on a sediment–rock transition zone. (c) Stony coral *Ellanopsammia rostrata* on bedrock within a sediment–rock transition zone, occasionally used as habitat by the orange roughy (*Hoplostethus atlanticus*). (d) Euplectellid glass sponge (*Regadrella* sp.) attached to bedrock partially covered by a thin sediment veneer. (e) Large branched primnoid corals (*Calyptrophora* sp.) on exposed bedrock. (f) Sponge field (Stelletta sp.) colonizing coral rubble derived from *Madrepora oculata*. The white scale bar represents 10 cm. Images are from Schmidt Ocean Institute [66].

When substrate type was examined independently within each dive, substrate effects were strong in both dives but were most pronounced in dive S0643 (Figs 7 and 8). Hard substrates were associated with typical hard-bottom suspension feeding taxa that depend on hard surfaces for attachment. Bedrock and bedrock with sediment veneer substrates both had high contributions from crinoids (Antedonidae), the corals *Hemicorallium* sp.*,* and *Chrysogorgia* sp. and urchins *Caenopedina* sp. (Fig 8). Some of these taxa were only associated with outcropping bedrock (*Solenosmilia* sp.*, Caryophyllia* sp.*,* Stolonifera sp.) or areas of bedrock with sediment veneers (*Acanella* sp.*, Bathypathes* sp.*, Nematocarcinus* sp.*, Aspidodiadema* sp) showing some differentiation in affinities for hard or semi-hard substrate. Areas of mixed (sediment–rock transition) substrate hosted primnoid corals *Calyptrophora* sp.*,* actiniarians (*Actinoscyphia* sp.)*,* and black corals (*Telopathes* sp.) but also some reef-building corals (*Solenosmilia* sp.)*.* Sedimented areas were not significantly associated with a distinct fauna, although crinoids (Antedonidae) and bamboo-corals *Acanella* sp. were also found associated with these soft substrates (Fig 8). In dive S0645, fewer taxa were resolved with distinct substrate signals. Areas of bedrock hosted gorgonians (Plexauridae spp.) and nematocarcinid shrimp that contributed to contrasts across habitat types. Bedrock with sediment veneer was distinguished mainly by *Chrysogorgia* sp. reflecting a shift in community composition with an increase observed coincident with sediment-veneered surfaces. The clearest substrate signal was associated with biogenic rubble. Across all substrate pairs involving biogenic rubble, the sponge *Stelletta* sp. showed the highest contributions, indicating a tight association with biogenic rubble. Whilst *Stelletta* sp. occurred on other substrates, its predominance on areas of biogenic rubble substrates shows a distinct sponge-assemblage association.

#### 3.3.4. Combined habitat-substrate associations.

The combined habitat-substrate SIMPER analyses (Figs 7 and 8) provided further insight into the association among habitat, substrate type and benthic assemblage composition. Overall, the combined analyses suggested that substrate modifies habitat level patterns in some habitats, however the relative importance of these factors differed with depth and may also reflect the influence of oceanographic conditions that we did not include in the present analyses. The number of taxa contributing significantly to the SIMPER also contrasted between dives, with S0643 consistently exhibiting a broader suite of contributing taxa than S0645.

In the deeper dive S0643, habitat explained most of the observed compositional differences, with some substrate-driven differences within habitat. In the shallower dive S0645, substrate type exerted a strong influence on assemblage composition across habitats, with habitat-level effects. In S0643, the most distinct assemblages occurred in the ridge crest (bedrock with sediment veneer, followed by bedrock and sediment–rock transition). These ridge crest assemblages were consistently characterised by high contributions from reef-building corals (*Solenosmilia* sp.*, Enallopsammia* sp.), solitary corals (*Caryophyllia* sp.), octocorals (*Calyptrophora* sp.*, Chrysogorgia* sp.), red coral (*Hemicorallium* sp.)*,* and malacalcyonacea (Malacalcyonacea sp.) (Fig 8). Within the ridge crest habitat changes in substrate type corresponded to changes in the relative contributions of framework-forming and branching suspension feeders. *Solenosmilia* sp. was a key discriminator between bedrock and sediment–rock transition or bedrock with sediment veneer. Solitary corals, primoids *Calyptrophora* sp. and *Chrysogorgia* sp. also contributed to the bedrock contrast, as do *Hemicorallium* sp. and Malacalcyonacea sp. On the S0643S0643 ridge slope habitat, contrasts among substrates were driven primarily by large suspension feeders and echinoderms. Substrates comprising bedrock with sediment veneer hosted the most diverse assemblage, followed by sediment–rock transition and finally areas of sediment. Whilst the majority of taxa observed on the ridge slope were linked to bedrock with sediment veneer, black coral *Telopathes* sp.*,* primnoid *Calypthrophora* sp.*,* octocoral *Acanella* sp. and actiniarian *Actinoscyphia* sp. were found across multiple substrate types (Fig 8).

In S0645, the combined habitat-substrate patterns were simpler but still revealed a few strong signals. In the S0645 ridge crest habitat, biogenic rubble was a uniquely structured macro-habitat supporting a distinct assemblage dominated by *Stelletta* sp. Differences between hard substrate macrohabitats (bedrock vs bedrock with sediment veneer) in the ridge crest areas were mainly driven by *Chrysogorgia* sp. which consistently had higher contributions in sediment veneer habitats. In the S0645 ridge slope habitat however, bedrock was characterised by Plexauridae and *Nematocarcinus* sp., differentiating slope from crest.

## 4. Discussion

### 4.1. Automated and quantitative habitat mapping

Deep-sea habitats are inherently hierarchical, with physical and biological processes operating across multiple spatial scales, from mega- to macro- and micro-scales that collectively shape habitat distributions, community composition, and ecosystem functioning [46–48,104,105]. Seamounts in particular exhibit environmental heterogeneity due to variations in topography, morphology, substrate, and hydrodynamics, creating distinct habitats that influence benthic assemblages [16,22,29,52,72]. A fundamental step in understanding these systems is the mapping and characterisation of seamount morphology and associated abiotic habitat structure [16, 17, 43, 47, 49, 106]. Traditional approaches including expert-driven morphological mapping and rule-based classifications such as the Benthic Terrain Modeler have been widely applied to characterize habitat classes of seamount and other benthic environments [41,56,107]. These methods provide valuable morphological insights but typically rely on user-defined thresholds, classification rules, and expert interpretation, which can introduce subjectivity and reduce reproducibility and comparability among studies. A more advanced supervised or semi-supervised learning approaches such as Random Forest [108,109], Support Vector Machines [110,111], or convolutional neural networks [112–115] could generate more robust quantitative habitat maps. These approaches can integrate ground-truth observations with acoustic and terrain data to produce consistent and scalable habitat maps. However, their application in deep-sea environments is often constrained by the limited spatial coverage and representativeness of ground-truth data. In this study, only two ROV transects were available, which did not span the full bathymetric and morphological range of Solito Seamount, limiting the feasibility of supervised classification approaches.

Given these constraints, this study adopted a hybrid, unsupervised, data-driven approach that balances reproducibility with limited ground-truth availability. The integration of multibeam bathymetry, terrain derivatives, and backscatter intensity enabled the unsupervised characterisation of benthic mesohabitats across Solito Seamount. PCA combined with unsupervised k-means clustering enabled the segmentation of the seamount into meso-scale habitat classes defined by distinct combinations of depth, slope, rugosity, and substrate reflectance (backscatter intensity) (Figs 3 and 4). Similar multivariate and unsupervised approaches have been successfully applied in submarine canyon and seamount environments [51,52,59], demonstrating their utility for objectively delineating morphological zones that may correspond to ecologically meaningful habitat classes. In this study, these habitats represent spatially coherent morphological units (Fig 4). When overlayed by ROV-based visual observations, the resulting classifications enabled explicit links between morphology, substrate type, and benthic assemblages (Figs 6 and 7), reinforcing their ecological relevance. Although k-means clustering provides a simple and reproducible framework for unsupervised classification, it is constrained by assumptions of cluster geometry and separability and may produce spatially fragmented or ecologically ambiguous classes [49,51,116]. Nevertheless, in data-limited deep-sea settings, it offers a transparent and objective first-order approximation of habitat structure. Overall, this study presents a pragmatic middle-ground between expert-driven classification and fully supervised machine learning. While the current unsupervised framework enables reproducible multi-scale habitat characterisation, future work should prioritise the collection of spatially representative biological and substrate observations [49,76,117,118]. This would enable the development of supervised or hybrid classification frameworks, improving the accuracy of habitat delineation, strengthening ecological interpretation, and supporting evidence-based marine spatial planning.

### 4.2. Habitat and substrate on the Solito Seamount

The habitat structure identified via the K-means clustering approach for Solito is primarily controlled by depth, slope gradient, and surface roughness and hardness (backscatter intensity). The organisation of the five habitats aligns closely with the morphology of a cone-shaped seamount, where ridge and valley systems radiate downslope from a relatively narrow summit, and steeper volcanic slope, transitioning progressively into the surrounding abyssal plain (Fig 4a) [119,120]. This configuration strongly influences substrate distribution. The limited summit area and higher relief restrict the accumulation of unconsolidated particles, resulting in widespread exposure of volcanic hard substrate around the summit area. Accordingly, ridge crest and ridge slope are dominated by exposed bedrock and sediment veneer, with only localized sediment accumulation in small topographic depressions and fracture-controlled pockets (Figs 5c and 5d). In contrast, sediment cover increases downslope as gradients decrease, with valleys acting as sediment accumulation zones where reduced slopes and topographic confinement favour sediment retention. This habitat and substrate organisation differs from the flat-topped, guyot-dominated seamounts of the NZ and S&G ridges, which are characterised by broad, flat summits, comparatively subdued relief, and laterally extensive sediment-covered platforms, resulting in proportionally less-developed slope environments [36,121].

Biogenic rubble represents a spatially limited but ecologically distinct substrate type on Solito, preferentially associated with shallower depths (dive S0645) and intermediate slopes (Figs 4 and 6). The biogenic rubble on Solito Seamount is derived primarily from framework-forming corals such as *Madrepora oculata* and bamboo corals, taxa widely recognized as ecosystem engineers that enhance seafloor heterogeneity and promote benthic diversity by creating a range of ecological niches [122,123]. Its occurrence across multiple habitats suggests that biogenic rubble distribution is not solely controlled by large-scale morphology, but rather reflects a combination of biological production, physical fragmentation of biogenic frameworks, and local hydrodynamic processes that govern downslope transport and retention [41,124–126]. As such, biogenic rubble may function as a transitional substrate between unconsolidated sediments and exposed bedrock [126–128], contributing additional structural complexity on Solito.

### 4.3. Benthic fauna on the Solito Seamount

Both ROV dives and analyses confirm that habitat complexity and substrate composition are strongly associated with variation in benthic megafaunal assemblages, supporting previous studies identifying these as important correlates of community composition [122,129–131]. However, geomorphology and substrate can covary with oceanographic and hydrographic variables such as dissolved oxygen concentration, water mass structure, and current regimes on seamounts [40,132,133]. Therefore, while the associations observed in this study suggest that morphological setting and substrate heterogeneity contribute to structuring benthic assemblages, the observed patterns across habitats likely reflect the combined influence of habitat characteristics and covarying oceanographic gradients operating across multiple spatial scales For example, in dive S0643, distinct differences in assemblage composition between ridge crest and slope were apparent. Here, bamboo coral *Acanella* sp. was more abundant on sediment-rich slopes (Figs 7 and 8), whereas Antedonidae crinoids were most abundant on exposed bedrock (Fig 7). Theaffinity of *Acanella* sp. for soft substrate consistent with observations for the congener *Acanella arbuscula*, which can occupy different substrate types [134], but is more commonly associated with soft-sediments [135]. Both bamboo corals and crinoids are suspension feeders that rely on suspended organic particles and pelagic prey for their nutrition [e.g., 136, 137]. A possible explanation for the higher abundance of these taxa on ridge slopes may be linked with locally accelerated currents, which facilitate the transport and/or resuspension of organic particles and zooplankton, contributing to an increase in the abundance of certain taxa [138,139].

The contrasting community patterns between the two dives further suggest that the relative importance of habitat and associated environmental gradients varies with depth on Solito. The deeper assemblages (S0643) are characterised by a comparatively diverse suite of megafaunal taxa, including reef-building scleractinians (*Solenosmilia* sp.*, Caryophyllia* sp.*, Enallopsammia* sp.)*,* octocorals (*Acanella* sp.*, Calyptrophora* sp.*, Chrysogorgia* sp.)*,* antipatharians, actiniarians (*Actinoscyphia* sp.), and echinoderms (Fig 7). The shallower assemblage (S0645) was dominated by a smaller number of taxa with community differences primarily driven by a few abundant or structurally dominant species (*Stelletta* sp.). Although the terrain metrics used in this study captured substantial habitat variability across the seamount, with the first two principal components explaining a large proportion of environmental variation (S2 Fig), they do not capture oceanographic processes that also vary across the seamount. In this study, the shallower assemblages observed on dive S0645 coincide with the ESSW (~50–600 m) above the lower boundary of the OMZ, whereas the deeper communities in dive S0643 occur within well-oxygenated AAIW (600–1,300 m) and PDW (~1,500–3,000 m) [65]. Oxygen availability and water mass structure are well-established environmental filters influencing deep-sea benthic diversity and community composition [140,141], including on seamounts, with increased species richness often observed below the OMZ boundary where reduced oxygen limitation and greater organic matter availability support more diverse benthic assemblages [132,133]. Consistent with this interpretation, complementary analyses of the Solito Seamount ecosystem identified depth, dissolved oxygen, temperature, and salinity as significant correlates of benthic community composition [41].

At a local scale, although habitat-level differentiation was weaker in the shallower dive S0645, substrate remained an important correlate of community composition. This suggests that local substrate characteristics provide distinct settlement opportunities that can influence species distributions (Fig 7). The ridge crest showed substantial variation of taxa among biogenic rubble, bedrocks, and bedrock with a sediment veneer. Changes in the abundance of the sponge *Stelletta* sp. were particularly important explaining the dissimilarity of benthic assemblages among substrate types, showing higher abundances on biogenic rubble, followed by areas of outcropping bedrock, with the lowest abundances associated with bedrock with sediment veneer (Fig 8f). Biogenic rubble is increasingly recognised as an ecologically important substrate. Sponges are predominant components of shallow biogenic rubble macrohabitats and are considered key organisms due to their role in rubble binding and stabilization processes, as well as their role in the energy transfer through food webs [126]. Moreover, several sponge species exhibit significant habitat preference for non-living substrates, preferring dead corals, unconsolidated biogenic rubble and calcium carbonate rocks [142]. In deep-sea environments, biogenic rubbles are known to enhance the metabolic activity of cold-water coral reefs, where diverse faunal (including sponges) and microbial communities recycle considerable amounts of particulate (phytodetritus) and dissolved organic matter [143]. Similar ecological roles associated with biogenic rubble have been reported from seamount JF6 and other seamounts of the Nazca–Desventuradas region, suggesting recurrent functionality roles across biogeographically connected systems [39,40].

The presence of shared habitat-forming taxa and commercially important species with the Nazca-Desventuradas and Juan Fernández systems, suggests that Solito may contribute to biogeographic stepping stone within the broader southeastern Pacific seamount network, potentially facilitating faunal connectivity across adjacent region [39,144]. The quantitative baseline established here provide an important foundation for future investigations of the interacting roles of meso-scale habtiat, substrate, and oceanographic processes in shaping deep-sea biodiversity and supports Solito Seamount as a priority candidate for inclusion in regional marine spatial planning and conservation strategies, particularly given its habitat heterogeneity and likely role in sustaining deep-sea connectivity.

## 5. Conclusion

This study uses an automated and objective habitat mapping approach with *in-situ* biological observations to resolve the morphological and ecological organisation of a deep-sea seamount. By combining high-resolution multibeam bathymetry and backscatter intensity data with ROV-based observations of substrate and megafauna, we performed a quantitative and reproducible analysis that links geomorphology, habitat structure, and benthic community patterns at Solito. The automated, multi-scale approach advances beyond traditional rule-based methods, provides the physical context necessary to interpret variation in megafaunal composition, abundance, and biodiversity. Solito supports diverse and structurally complex benthic communities dominated by cnidarians, echinoderms, sponges, and arthropods. The distribution and composition of benthic megafauna on Solito are strongly correlated to habitat type and substrate heterogeneity. Community differences between dives highlight the interaction of habitat complexity and broader-scale depth associated environmental gradients in shaping assemblages below the oxygen minimum zone. With spatial limitations of ROV surveys, this study outlines a pathway toward more robust, and transferable deep-sea habitat mapping approach. More broadly, integrative frameworks such as the one presented here provide essential baselines for biodiversity assessment, spatial planning, and the conservation of vulnerable seamount ecosystems, particularly in regions facing increasing anthropogenic pressure and limited biological knowledge.

## Supporting information

S1 Fig Bathymetry and terrain derivatives used to map the habitats of the Solito Seamount. (a) Depth, (b) Backscatter intensity, (c) Slope, (d) Broad-scale BPI (bathymetric position index), (e) Fine-scale BPI, (f) Planform curvature (g) Profile curvature, (h) TRI (terrain ruggedness index) (i) VRM (vector ruggedness measure).
(TIF)

S2 Fig Scree plot of principal component analysis (PCA) showing the proportion of variance explained by individual components (green bars) and the cumulative variance explained (blue line), with a 90% cumulative variance threshold indicated. (TIF)

S3 Fig Violin plots showing the distribution of standardized (Z-score) terrain variables across habitat classes. BPI: bathymetric position index; TRI: terrain ruggedness index; VRM: vector ruggedness measure. Each violin represents the density distribution of values within a habitat class, with an embedded boxplot. In the boxplots, the centre line indicates the median, the lower and upper box boundaries represent the first and third quartiles (interquartile range), and the whiskers extend to the most extreme values within 1.5 × IQR. Points beyond the whiskers denote outliers.
(TIF)

S1 Table List of multibeam bathymetry and backscatter intensity derivatives. (DOCX)

S2 Table A principal component analysis was conducted to reduce data dimensionality and compute a set of new and linearly independent abiotic variables. Five principal components (PCs), which explained 93% of the total variance in the data, were retained for subsequent analysis. See S1 Table for definition of acronyms.
(DOCX)

S3 Table Results of nested PERMANOVA testing differences in assemblage structure. Df: Degrees of Freedom, SS: Sum of Squares, MS: Mean Square.
(DOCX)

S4 Table Pairwise PERMANOVA comparisons among habitats within each dive. (DOCX)

S5 Table Pairwise PERMANOVA comparisons among substrates within habitat and dive. Substrate 1 = bedrock, 2 = sediment veneer, 3 = sediment-rock transition, 4 = sediment, 5 = biogenic rubble.
(DOCX)
