# Supplementary material for: Automated and quantitative characterization of benthic habitat and associated biological communities of an unknown southeast Pacific seamount
Source: PLoS One. 2026 Sep 15;21(9):e0345687. doi: 10.1371/journal.pone.0345687 (PMC13577503; doi:10.1371/journal.pone.0345687)
Supplement: Supplementary file 1 [file pone.0345687.s004.docx]

S1 Table. List of multibeam bathymetry and backscatter intensity derivatives.

| **Variable name** | **Units** | **Description** |
| --- | --- | --- |
| Depth | m | Vertical distance below sea level (bathymetry). |
| Backscatter | dB | Local standard deviation of backscatter; reflects texture variability. |
| Slope | ° or radians | Gradient or rate of maximum change in z-value; represents terrain steepness. |
| Bathymetric Position Index (BPI) | Unitless | Relative position of a cell compared to surrounding cells; positive = ridge, negative = depression. |
| Curvature (Planform) | m⁻¹ × 100 | Curvature perpendicular to slope; affects flow convergence/divergence. |
| Curvature (Profile) | m⁻¹ × 100 | Curvature parallel to slope; influences acceleration/deceleration of flow. |
| TRI (Terrain Ruggedness Index) | m | Mean absolute elevation difference between focal cell and neighbours. |
| VRM (Vector Ruggedness Measure) | Unitless | Dispersion of surface normal vectors; integrates slope and aspect variability. |
