# Supplementary material for: Automated and quantitative characterization of benthic habitat and associated biological communities of an unknown southeast Pacific seamount
Source: PLoS One. 2026 Sep 15;21(9):e0345687. doi: 10.1371/journal.pone.0345687 (PMC13577503; doi:10.1371/journal.pone.0345687)
Supplement: Supplementary file 2 [file pone.0345687.s005.docx]

S2 Table. A principal component analysis was conducted to reduce data dimensionality and compute a set of new and linearly independent abiotic variables. Five principal components (PCs), which explained 93% of the total variance in the data, were retained for subsequent analysis. See S1 Table for definition of acronyms.

|  | RC1 | RC2 | RC3 | RC4 | RC5 | RC6 | RC7 |
| --- | --- | --- | --- | --- | --- | --- | --- |
| Depth | -0.0019 | 0.5679 | -0.0114 | 0.0436 | 0.0749 | 0.8184 | 0.0051 |
| Backscatter | -0.0088 | 0.0336 | 0.9978 | -0.0026 | 0.0563 | -0.0061 | -5.00E-04 |
| Slope | 0.0262 | 0.9744 | 0.0192 | -0.0318 | 0.113 | 0.1777 | 0.0068 |
| Broad-BPI | 0.8012 | 0.0426 | -0.006 | 0.3798 | 0.229 | 0.0209 | 0.3945 |
| Fine-BPI | 0.8415 | 0.0211 | -0.0059 | 0.4703 | 0.1582 | 0.0176 | 0.1904 |
| Curvature (Planform) | 0.9663 | 0.0585 | -0.0039 | -0.0075 | 0.0407 | -0.0154 | -0.2455 |
| Curvature (Profile) | 0.2772 | -0.038 | -0.002 | 0.9571 | 0.0638 | 0.029 | 0.0171 |
| TRI | 0.0644 | 0.9563 | 0.034 | -0.0103 | 0.2211 | 0.1633 | -0.0013 |
| VRM | 0.197 | 0.2917 | 0.0702 | 0.0877 | 0.9271 | 0.0574 | 0.0257 |
