# Supplementary material for: Automated and quantitative characterization of benthic habitat and associated biological communities of an unknown southeast Pacific seamount
Source: PLoS One. 2026 Sep 15;21(9):e0345687. doi: 10.1371/journal.pone.0345687 (PMC13577503; doi:10.1371/journal.pone.0345687)
Supplement: Supplementary file 3 [file pone.0345687.s006.docx]

S3 Table. Results of nested PERMANOVA testing differences in assemblage structure. Df: Degrees of Freedom, SS: Sum of Squares, MS: Mean Square.

| Source | Df | SS | MS | Pseudo-F | P(perm) | Unique permutations |
| --- | --- | --- | --- | --- | --- | --- |
| Dive (random) | 1 | 7864.3 | 7864.3 | 2.0205 | 0.008 | 998 |
| Habitat/Dive | 4 | 25595 | 6398.8 | 1.644 | 0.001 | 998 |
| Substrate/Habitat/Dive | 9 | 76784 | 8531.6 | 2.192 | 0.001 | 996 |
| Residual | 118 | 4.59E+05 | 3892.2 |  |  |  |
| Total | 132 | 6.07E+05 |  |  |  |  |
