# Supplementary material for: Automated and quantitative characterization of benthic habitat and associated biological communities of an unknown southeast Pacific seamount
Source: PLoS One. 2026 Sep 15;21(9):e0345687. doi: 10.1371/journal.pone.0345687 (PMC13577503; doi:10.1371/journal.pone.0345687)
Supplement: Supplementary file 4 [file pone.0345687.s007.docx]

S4 Table. Pairwise PERMANOVA comparisons among habitats within each dive.

| Dive ID | Habitat comparison | t | P(perm) |
| --- | --- | --- | --- |
| S0643 | Ridge crest vs ridge slope | 1.399 | 0.005 |
| S0645 | Ridge crest vs ridge slope | 1.269 | 0.112 |
|  | Ridge slope vs basal slope | 1.137 | 0.333 |
|  | Ridge slope vs valley | 1.096 | 0.333 |
|  | Ridge crest vs basal slope | 1.294 | 0.136 |
|  | Ridge crest vs valley | 1.344 | 0.055 |
|  | Basal slope vs valley | – | – |
