# Supplementary material for: Automated and quantitative characterization of benthic habitat and associated biological communities of an unknown southeast Pacific seamount
Source: PLoS One. 2026 Sep 15;21(9):e0345687. doi: 10.1371/journal.pone.0345687 (PMC13577503; doi:10.1371/journal.pone.0345687)
Supplement: Supplementary file 5 [file pone.0345687.s008.docx]

S5 Table. Pairwise PERMANOVA comparisons among substrates within habitat and dive. Substrate 1 = bedrock, 2 = sediment veneer, 3= sediment-rock transition, 4 = sediment, 5=biogenic rubble.

| Dive ID | Habitat | Substrate comparison | t | P(perm) |
| --- | --- | --- | --- | --- |
| S0643 | Ridge slope | 1 vs 2 | 1.121 | 0.166 |
|  |  | 1 vs 3 | 1.259 | 0.044 |
|  |  | 1 vs 4 | 2.409 | 0.008 |
|  |  | 2 vs 3 | 1.097 | 0.179 |
|  |  | 2 vs 4 | 2.092 | 0.001 |
|  |  | 3 vs 4 | 1.951 | 0.010 |
|  | Ridge crest | 1 vs 2 | 1.279 | 0.060 |
|  |  | 1 vs 3 | 1.021 | 0.370 |
|  |  | 2 vs 3 | 1.205 | 0.090 |
| S0645 | Ridge slope | 1 vs 5 | 1.208 | 0.150 |
|  | Ridge crest | 1 vs 2 | 1.230 | 0.138 |
|  |  | 1 vs 5 | 2.414 | 0.001 |
|  |  | 2 vs 5 | 1.516 | 0.031 |
|  | Valley | 1 vs 5 | – | – |
